# Transcriptome analysis of high- and low-selenium genotypes identifies genes responsible for selenium absorption, translocation, and accumulation

**DOI:** 10.3389/fpls.2024.1413549

**Published:** 2024-09-23

**Authors:** Ling Li, Muhammad Zahir Ahsan, Zhe Li, Faiz Hussain Panhwar, Yue Zhang, Dan Luo, Yang Su, Xiaomei Jia, Xiaoying Ye, Caihong Shen, Songtao Wang, Jianqing Zhu

**Affiliations:** ^1^ Rice Research Institute, Sichuan Agricultural University, Chengdu, Sichuan, China; ^2^ National Engineering Research Center of Solid-State Brewing, Luzhou, Sichuan, China; ^3^ Demonstration Base for International Science & Technology Cooperation of Sichuan Province, Chengdu, Sichuan, China

**Keywords:** selenium, rice, transcriptome, WGCNA, hub genes

## Abstract

**Introduction:**

Selenium is an essential micronutrient the human body requires, which is closely linked to health. Rice, a primary staple food globally, is a major source of human selenium intake. To develop selenium-enriched rice varieties, it is imperative to understand the mechanisms behind selenium’s absorption and transport within rice, alongside identifying the key genes involved in selenium uptake, transport, and transformation within the plant.

**Methods:**

This study conducted transcriptome sequencing on four types of rice materials (two with low-selenium and two with high-selenium contents) across roots, stems, leaves, and panicles to analyze the gene expression differences.

**Results and discussion:**

Differential gene expression was observed in the various tissues, identifying 5,815, 6,169, 7,609, and 10,223 distinct genes in roots, stems, leaves, and panicles, respectively. To delve into these differentially expressed genes and identify the hub genes linked to selenium contents, weighted gene co-expression network analysis (WGCNA) was performed. Ultimately, 10, 8, 7, and 6 hub genes in the roots, stems, leaves, and panicles, respectively, were identified. The identification of these hub genes substantially aids in advancing our understanding of the molecular mechanisms involved in selenium absorption and transport during the growth of rice.

## 1 Introduction

Selenium is an essential micronutrient for the human body and is a component of 25 functional proteins, including glutathione peroxidase (GSH-Px) enzyme, having antioxidant capacity 200 times higher than that of vitamins (Taneja, 2016). In addition, selenium plays an important role in enhancing the body’s immune system and fighting against diseases such as cancer, cardiovascular disease, and diabetes (Rayman et al., 2008; Benstoem et al., 2015; Cai et al., 2016; Tan et al., 2016). In recent years, the problem of hidden hunger due to selenium deficiency has become increasingly serious worldwide (Kumar et al., 2016; D'Amato et al., 2018; Gao et al., 2018), particularly in Asian countries with major rice-producing regions. The selenium intake by residents in these areas is significantly lower than the recommended level of the European Food Safety Authority (EFSA) (D'Amato et al., 2018; Gao et al., 2018). Plants first absorb selenium from the soil, then absorbed and utilized by humans in the form of food. So the total selenium intake by humans is mainly determined by the soil selenium contents and available selenium in the plants (Malagoli et al., 2015). However, with time, the selenium-deficient areas worldwide exceed the high selenium areas (White et al., 2012). Rice (*Oryza sativa* L.), the major food crop in Asia, provides up to 80% energy, protein, and trace elements and is also one of the main sources of selenium intake. The human selenium level in these areas is closely related to the selenium contents of rice (Tang and Cheng, 2018). In recent years, selenium-rich food commodities, especially the production of high-selenium rice, have provided an important way for selenium-deficient people to supplement selenium (Wan et al., 2018). Therefore, increasing the selenium contents in rice grains is the safest and most effective way to supplement human selenium in these regions.

In rice production, the application of selenium fertilizer can effectively increase the selenium contents in rice grains and, at the same time, promote growth, yield, nutrition and appearance quality, reduce heavy metal toxicity, and enhance stress resistance (Malagoli et al., 2015). The selenium contents in plants mainly come from the soil by root absorption, and the selenium accumulation in rice tissues and grains gradually increases with the extension of the rice growth period. The selenium contents in rice tissues at the maturity stage are high and in the following order: roots> leaves> stem> panicle (Broadley et al., 2006). Selenium exists in different forms in soil, among which selenate and selenite are water-soluble selenium, which are relatively easy for plants to absorb and utilize (Neal and Sposito, 1989). Selenate is more common and bioavailable than selenite in oxidizing and alkaline soils, while selenite is more common in neutral or acidic anaerobic soils (White et al., 2007).

Phosphate and sulfate transporters in millets are responsible for selenium uptake (Mushtaq et al., 2022). Selenate (SeO_4_^2−^) is the most common form of selenium that plants absorb and utilize (Mushtaq et al., 2022). It is a chemical analog of sulfate (SO_4_^2−^) and enters the plant by competing sulfate for sulfur transporters (SULTR) on the cell membrane (White et al., 2004; Huang et al., 2015). Unlike the absorption pathway of selenate, selenite (SeO_3_^2−^) absorption is carried out through the phosphorus transport pathway (Li et al., 2008). Selenite shares the same protein channel as phosphate, and they have a competitive absorption relationship. In rice, overexpression or knockdown of phosphate transporter OsPT2 in roots significantly affected selenite uptake in roots (Zhang et al., 2014). Selenite uptake in roots decreases when OsPT2 is overexpressed, and its uptake increases when OsPT2 is knocked down suggesting that there is uptake competition between selenite and phosphate, and these two molecules share a common transporter (Lazard et al., 2010). Regardless of the valence state in which plants absorb selenium, its metabolism is done through the sulfur metabolism pathway, which converts selenium into selenosylated amino acids, an organic selenium (Pilon-Smits et al., 2014). After the plant roots absorb selenate, it combines with ATP in the cytoplasm or plastid to form adenosine 3′-phosphate 5′-phosphoryl selenate (APSe), which is reduced to selenite by adenosine phosphate (APS) reductase that is converted to Se^2−^ under the action of selenite reductase or reduced glutathione. Finally, this Se^2−^ through replication and cumbersome metabolism, is reduced to organic selenium and transported to other plant organs (Schiavon et al., 2015).

Selenium is chemically similar to sulfur, and in plants, selenate might be absorbed and utilized with the help of sulfate transporters (Howarth et al., 2003). The selenium content of *Arabidopsis* plants was significantly reduced by knocking out the sulfate transporter gene *sultr1:2* in *Arabidopsis thaliana* (Kassis et al., 2007). By studying the spatial distribution characteristics of selenium in the leaves, stems, roots, and seeds, it was found that selenium was similar to sulfur indicating that selenium uptake was related to sulfur transporters (Pickering et al., 2000). However, the pathways of selenium absorption, transport, and accumulation between different tissues in the plant body and the genes for selenium synthesis remain unclear. Therefore, how selenium is absorbed, accumulated, and distributed in plants still needs further study.

Transcriptome sequencing and weighted correlation network analysis (WGCNA) have been widely used to analyze plants’ molecular mechanisms, gene expression, and key gene networks of various physiological and biochemical processes (Langfelder and Horvath, 2008). WGCNA is an R package for weighted correlation network analysis (Langfelder and Horvath, 2008). It can be used for finding clusters (modules) of highly correlated genes, for summarizing such clusters using the module eigengene or an intramodular hub gene, and for relating modules to one another and to external sample traits. This method has been successfully applied to soybeans, tomatoes, cucumbers, and other crops to identify gene modules and core genes that are highly involved in plant growth, development, and physiological processes (Wei et al., 2016; Qi et al., 2018; Zou et al., 2018). In rice, WGCNA was used to analyze the transcriptome data of drought, low temperature, salt stress, and low nitrogen and to identify the core genes of these traits and the modules of co-expressed genes under these stress conditions (Zhu et al., 2019; Wang et al., 2023; Zeng et al., 2022). However, there is still a lack of research on the absorption, transport, morphological transformation, and accumulation of selenium in rice plants. Therefore, transcriptome sequencing and WGCNA can be used to explore these horizons.

Rice grains is an important storage organ for selenium, and the organic Se species SeMet and SeMeSeCys are rapidly loaded into the phloem and transported to the grain during grain fill [Sun et al. (2010). Distribution and Translocation of Selenium from Soil to Grain and Its Speciation in Paddy Rice (*Oryza sativa* L.). *Environ. Sci. Technol.* 44(17), 6706–6711. doi:10.1021/es101843x; Carey et al. (2012). Grain Accumulation of Selenium Species in Rice (Oryza sativa L.). *Environ. Sci. Technol.* 46(10), 5557–5564. doi:10.1021/es203871j]. SeMet was the main type of selenium in rice grains, which accounted for 82.9% of total selenium (Sun et al., 2010). In wheat, SeMet is transferred to the ear during the grain-filling stage and greatly increased the total selenium content (Fabienne et al., 1999). During grain-filling stage, the organic selenium (SeMet) is rapidly loaded into the phloem and transported to the grains more efficiently than inorganic selenium species (Carey et al., 2012). Therefore, transcriptome sequencing and WGCNA were performed on the roots, stems, leaves, and panicles at the grain-filling stage of high- and low-selenium rice genotypes to screen the core genes for selenium uptake, transport, and accumulation. This study aimed to explore the selenium absorption, transport, and accumulation-related genes that regulate selenium-related biosynthesis pathways in rice plants. The results will provide the foundation for understanding the biosynthesis and regulation of selenium absorption, transport, and accumulation in high- and low-selenium genotypes.

## 2 Materials and methods

### 2.1 Experimental materials and plant cultures

The materials used in this study were two low-selenium-content genotypes, Chenghui 727 (CH727: X3) and Yuenong Silk Miao (YNSM: X4), and two high-selenium-content genotypes, 2057B (X5) and 5097B (X8), and six repeats were set for each treatment.

Surface soil of 0–20 cm in the experimental field of Sichuan Agricultural University was collected and then dried by natural air. After grinding, the soil was screened by 3 mm and mixed for later experiments. In this experiment, 30 cm × 30-cm plastic pots were used to pack soil, and 6 kg of air-dried soil was filled in each pot. Three rice seedlings were planted in each pot. The experiment was conducted in the experimental field of the Rice Research Institute of Sichuan Agricultural University (Wenjiang Campus, Chengdu), and the whole growth period was 128 days. The basic physicochemical properties of the soil were as follows: pH 6.10, organic matter 29.35 g/kg, total nitrogen 0.201 g/kg, total phosphorus 1.59 g/kg, total potassium 12.1 g/kg, and total selenium 0.31 mg/kg.

Six replicated samples from roots, stems, leaves, and ears of testing genotypes were taken at the grain-filling stage. After repeated rinsing with deionized water, three replications were cryopreserved with liquid nitrogen for transcriptome sequencing, and the other three replicates were dried at 80°C, crushed, and passed through a 100-mm mesh sieve for subsequent selenium content determination.

### 2.2 Selenium content determination

According to the preceding method (Liang et al., 2018), samples (0.1 g) were weighed, placed in a glass vial, and then digested with 10 ml of HNO_3_/HClO_4_ mixed acid solution (9:1, v/v) on a 180°C electric hot plate (EH20A Plus, Labtech, USA) until 1 ml of whitish solution was generated. According to the previous method (Farooq et al., 2019), the digested samples were diluted by 10 ml of HCl/H_2_O solution (1:1, v/v), and then, the solution was digested at 120°C until 1 ml of whitish solution was again generated. The collected solution was made up to a volume of 10 ml with a 5% HCl solution in a centrifuge tube, which was analyzed by an atomic fluorescence spectrophotometer (RGF-6800, Bo Hui Co., Ltd., Beijing, P.R. China).


$$\text{Se content}=\frac{\left(C-C_{0}\right)\times V\times 1000}{M\times 1000\times 1000}$$

The Se content was expressed as mg/kg, where C is the measured Se concentration of the digestive solution (ng/ml), C_0_ is the Se concentration of the control group (ng/ml), M is the mass of the sample, and V is the total volume of the digestion solution. The analysis was repeated in triplicate.

### 2.3 RNA extraction and quantitative analysis of core genes

At the grain-filling stage, three replicated samples from four experimental materials were taken from the roots, stems, leaves, and panicles for RNA extraction. The total RNA was extracted as described in the RNA isolation kit manual (Boer, Chongqing, China). Equal amounts of RNA from three biological replicates were used to construct RNA libraries and transcriptome libraries. The quality and quantity of the sample libraries were examined using an Agilent 2100 Bioanalyzer and Nanodrop2000. First-strand cDNA was synthesized from 1 µg of total RNA using the PrimerScriptTM RT reagent kit with gDNA Eraser (Takara, Dalian, China).

### 2.4 Transcriptome sequencing and analysis

The extracted RNA was constructed into a nucleic acid library and sequenced by Prima (Shanghai, China) using the Illumina HiSeq-PE150 platform to obtain the raw data. Low-quality data was filtered using Trimmomatic (v0.39) software (Bolger et al., 2014). The filtered data were aligned to the rice reference genome by Hisat2 (V2.0.5) (Kim et al., 2019). The reads were counted using HTseq (v0.7.2), and the expression of fragments per kilobase per million read (FPKM) values of each gene was converted by Cufflinks (v2.2.1b) (Trapnell et al., 2012). To screen differentially expressed genes (DEGs) between the high- and low-selenium genotypes, the DESeq2 R package (v1.18.0) was used (Love et al., 2014). When the expression level of the same gene between different genotypes was fold change >2, FDR <0.05, the gene was considered to be differentially expressed.

The differentially expressed genes between high- and low-selenium genotypes in each tissue and the selenium contents of the four genotypes were used for WGCNA. WGCNA was performed using the R package “WGCNA.” One-step network construction was used to construct gene system tree diagrams, and the correlation between modules and selenium contents was analyzed. The parameters of the network build were set to tom-Type=unsigned, minimum module size = 30, and branch merge cut height = 0.25. The threshold power in the roots, stems, leaves, and panicles was set to 12, 16, 16, and 12, respectively.

### 2.5 Validation of deferentially expressed genes by qRT-PCR

The expression level of deferentially expressed genes, which showed significant changes in the transcriptional expression data, was screened by qRT-PCR analysis. Gene-specific primers were designed using Primer v5.0 and listed in **Supplementary Table S1**. qRT-PCR was performed using 20 μl (1 mM each primer, 0.1 mM cDNA, 10 μl of 2× SYBR Green Master mix) in three technological replicates on a qTOWER Real-Time PCR System (Analytik Jena, Germany). The relative expression of the genes was calculated using the 2–^ΔΔCt^ method (Livak and Schmittgen, 2001). *OsACTIN* (Os04g0177600) was used as an internal reference. The PCR conditions were as follows: primary denaturation at 95°C for 20 s followed by 40 amplification cycles of 3 s at 95°C and 30 s at 60°C. Each analysis was repeated three times.

### 2.6 Statistical analysis

Heatmap and principal component analysis (PCA) were analyzed and plotted using the “heatmap,” “factoextra,” and “psych” packages in R. GraphPad Prism 8.0.2 (https://www.graphpad.com) was utilized to perform statistical analysis. All statistical tests were two-tailed Student’s t-test at the 0.05 and 0.01 levels, and the level significance was given by the symbols * (p < 0.05) and ** (p < 0.01).

## 3 Result

### 3.1 Detection and analysis of selenium contents in different organs of rice at the grain-filling stage

**Figure 1:**
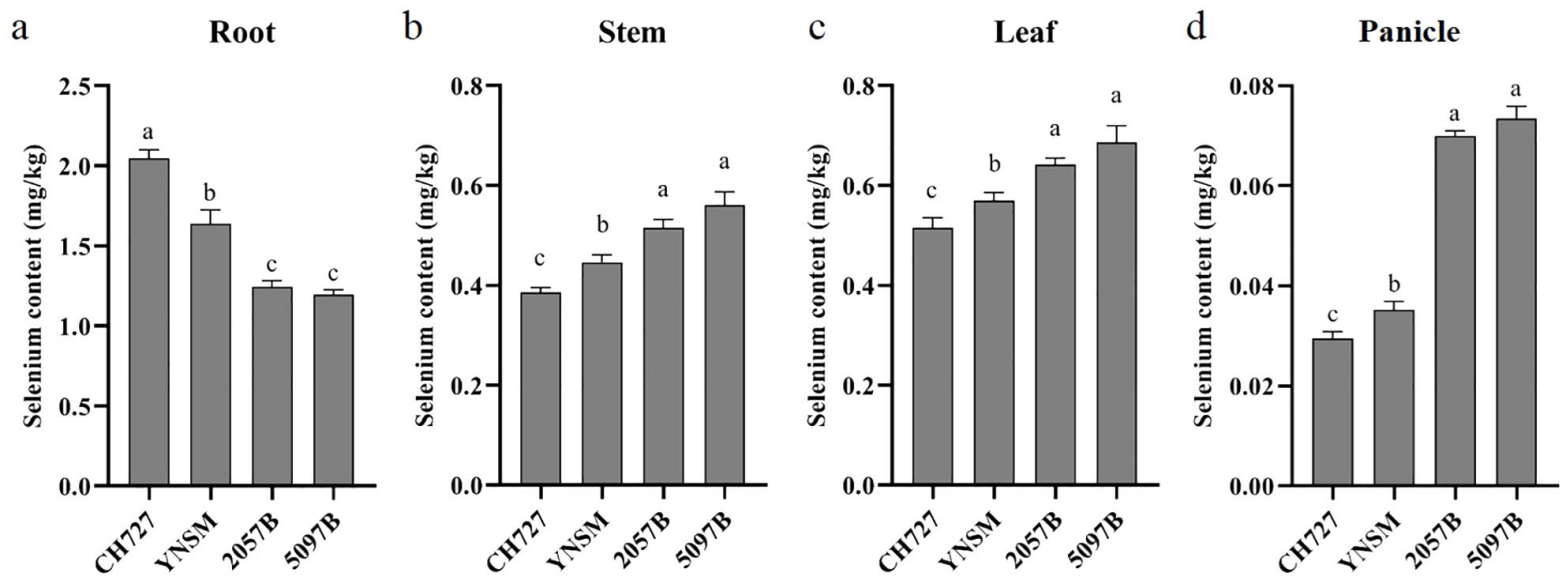
Comparison of selenium contents of four rice genotypes in the roots, stems, leaves, and panicles. **(A–D)** represent the selenium contents in roots, stems, leaves, and panicles, respectively. Different letters (a, b and c) on bars indicate the level of significance at p < 0.05.

The selenium contents in low-selenium (CH727 and YNSM) and high-selenium (2057B and 5097B) genotypes in the roots, stems, leaves, and panicles were tested at the grain-filling stage. The highest selenium contents were found in the roots of all genotypes, followed by the leaves, stems, and panicles (**Figure 1**).

Further, we compared the selenium contents in each tissue between genotypes. In the roots, the selenium contents of low-selenium genotypes were 2.05 and 1.64 mg/kg in CH727 and YNSM, respectively, which was significantly (p < 0.05) higher than that of high-selenium genotypes in 2057B and 5097B with 1.25 and 1.19 mg/kg, respectively (**Figure 1A**). In the stems, the selenium contents of low-selenium genotypes were 0.39 and 0.45 mg/kg in CH727 and YNSM, which was significantly (p < 0.05) lower than that of high-selenium genotypes in 2057B and 5097B with 0.52 and 0.56 mg/kg, respectively (**Figure 1B**). In the leaves, the selenium contents of low-selenium genotypes were 0.52 and 0.56 mg/kg in CH727 and YNSM, respectively, which was significantly (p < 0.05) lower than that of high-selenium genotypes in 2057B and 5097B with 0.64 and 0.69 mg/kg, respectively (**Figure 1C**). The difference in selenium contents in the panicles was particularly highly significant, exhibited ~50% high contents in high-selenium genotypes with 0.07 mg/kg compared to the low-selenium genotypes with 0.03 mg/kg (**Figure 1D**).

### 3.2 Transcriptome sequencing analysis of contrasting genotypes

**Figure 2:**
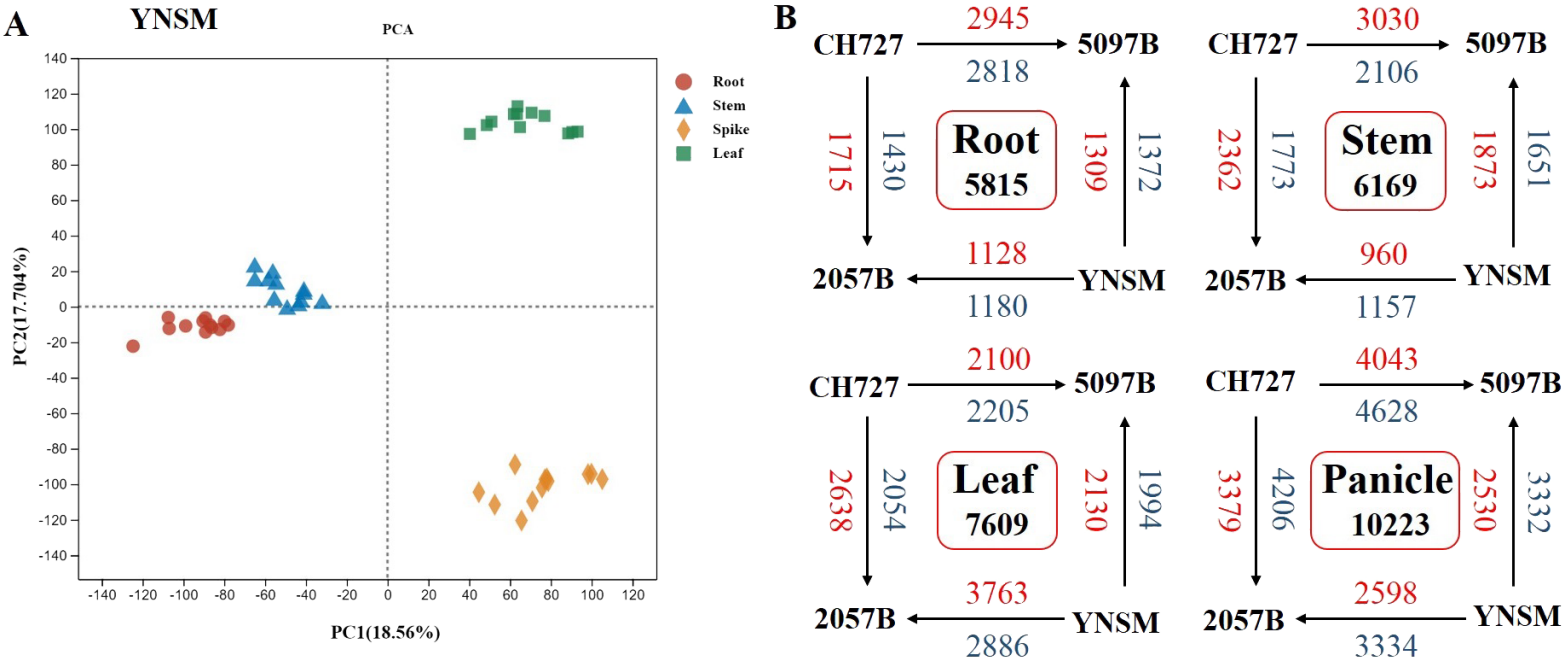
Overview of the differences in gene expression between four rice genotypes in the roots, stems, leaves, and panicles. **(A)** Principal component analysis of gene transcriptional profiles. **(B)** Number of differentially expressed genes (DEGs) between four rice genotypes in the roots, stems, leaves, and panicles (upregulated genes, red font; downregulated genes, blue font).

The transcriptome sequencing analysis was performed on four (2057B, 5097B, CH727, and YNSM) genotypes: root, stem, leaf, and panicle samples to explore the genes involved in selenium uptake and transport during rice growth. Finally, 2,315.98 million raw reads were obtained from transcriptome sequencing libraries after filtering low-quality data; 2,201.33 million reads were obtained with an effective utilization rate of 95.04% (**Supplementary Table S2**). The filtered data were compared with the reference rice genome; the proportion of each sample with genome was 86.92%–95.71%, with an average proportion of 92.50%. By analyzing the expression levels of genes in various tissues, 25,510, 24,553, 24,393, and 25,817 genes were expressed in the roots, stems, leaves, and panicles, respectively. In addition, from the principal component analysis (PCA) diagram, it could be found that the tissues of the roots, stems, leaves, and panicles can be divided into four groups, and the consistency of the distribution of biological replicates in each group indicated the consistency between sampling and experiment (**Figure 2A**). Through comparative analysis, it was found that the most differential expressed genes were identified in the panicles (10,223), followed by the leaves (7,609), stems (6,169) and roots (5,815) (**Figure 2B**). After removing the redundant genes in each tissue, the non-redundant differential expressed genes identified in the roots, stem, leaves, and panicles were analyzed by WGCNA to evaluate the correlation between each module and selenium contents and to identify the core genes involved in selenium absorption or translocation.

### 3.3 Identification of WGCNA modules related to selenium contents

**Figure 3:**
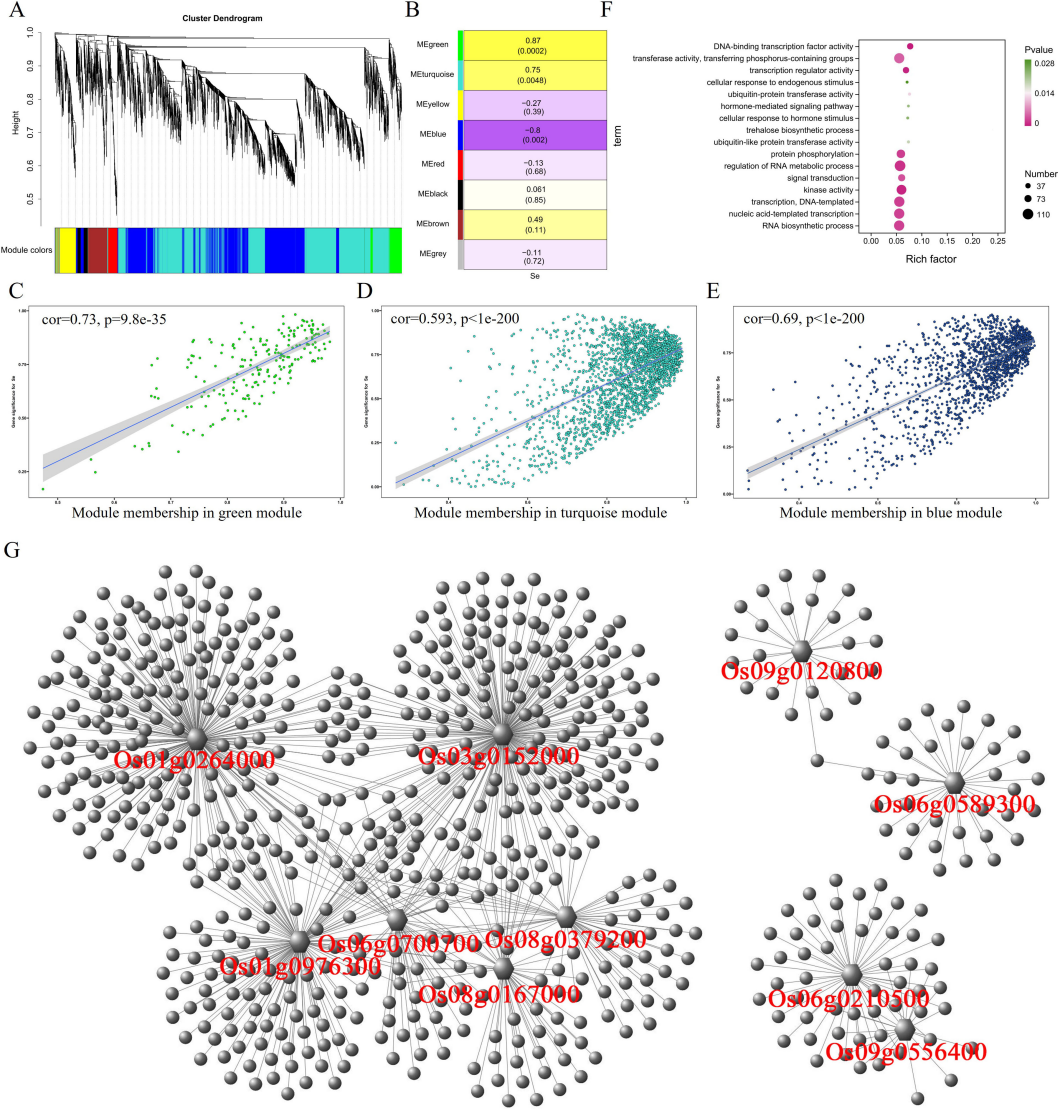
Weighted gene coexpression network analysis of differentially expressed genes with selenium contents in the root. **(A)** Hierarchical cluster tree showing eight modules with coexpressed genes. **(B)** Module with selenium content correlations and the corresponding p-values. **(C–E)** Correlation between module membership and significance of gene related to selenium contents. **(F)** GO annotation of genes in green, turquoise, and blue modules. **(G)** Network analysis of hub genes in the root.

In the roots, WGCNA analysis divided 5,815 differential expressed genes into eight modules (**Figure 3A**), and the correlation analysis between these eight modules and traits showed that MEgreen (r = 0.87, p = 0.0002) and MEturquoise (r = 0.75, p = 0.0048) had significant positive correlation with selenium contents, but MEblue module (r = −0.8, p = 0.002) was negatively correlated with selenium contents (**Figure 3B**). MEgreen, MEturquoise, and MEblue contained 201, 2,240, and 1,523 genes, respectively. The genes in the MEgreen module were significantly more expressed in the low-selenium genotypes (CH727 and YNSM) than in the high-selenium genotypes (2057B and 5097B) (**Supplementary Figure S1A**). The expression of MEturquoise module genes in CH727 was significantly higher than that of the other three materials, and the expression level of genes in 5097B was significantly lower than that of the other three genotypes (**Supplementary Figure S1B**). In the MEblue module, the gene expression in low-selenium genotype (CH727) was significantly lower than those of the other three genotypes (**Supplementary Figure S1C**). Further analysis showed that there was a significant correlation between module membership (MM) and gene significance (GS) in the three modules (**Figures 3C–E**). GO enrichment analysis of the genes in these three modules showed the following: GO:0016772: “transferase activity, transferring phosphorus-containing groups”; GO:0015706: “nitrate transport”; GO:0008272: “sulfate transport”; and other classes related to ion absorption and transport (**Figure 3F**; **Supplementary Table S3**). Some of these genes have been reported to be involved in selenium uptake and transport in rice, such as phosphate transporters Os06g0210500 and Os09g0454600 and sulfate transporters such as Os03g0195800, Os01g0719300, and Os08g0101500.

**Figure 4:**
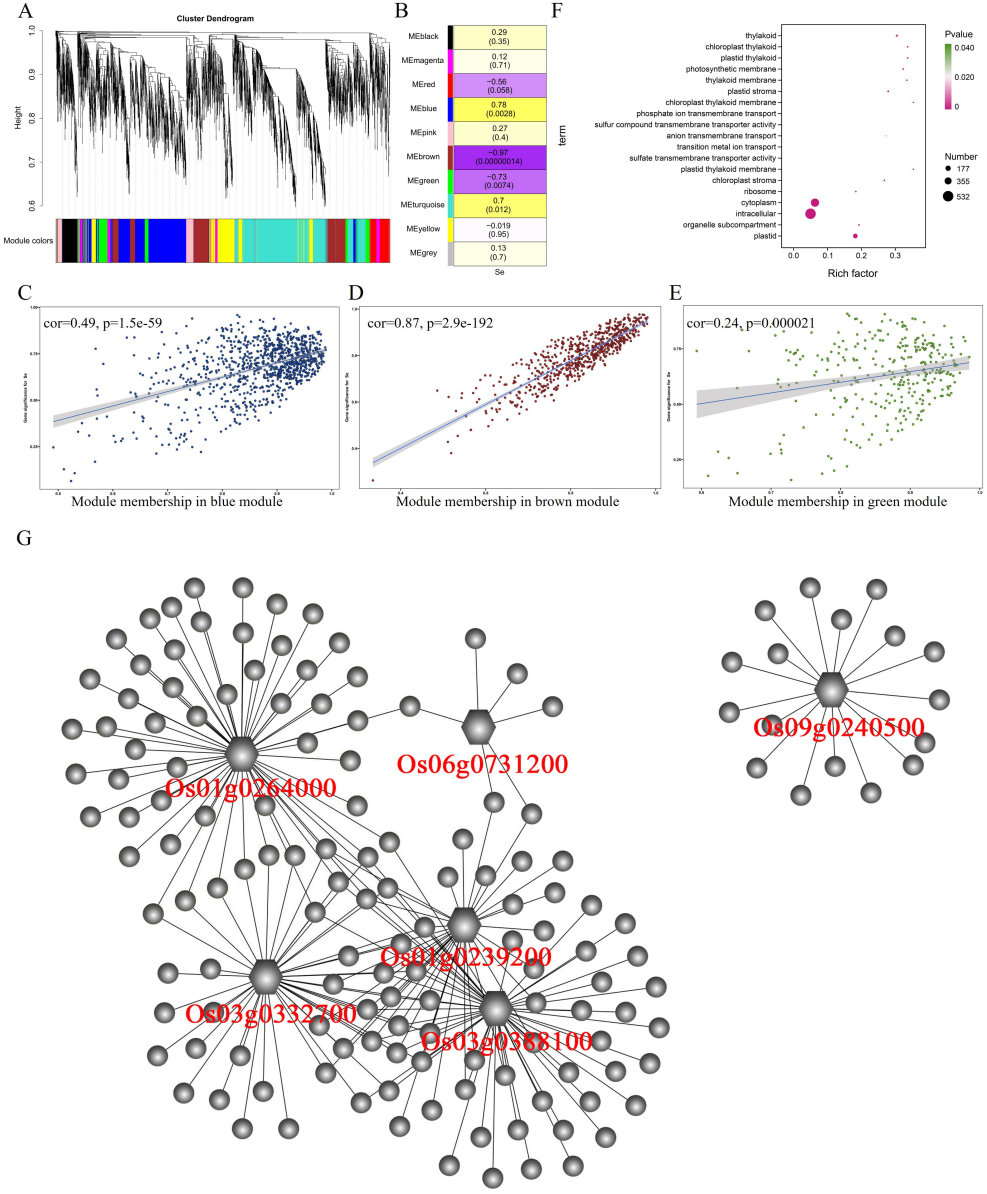
Weighted gene coexpression network analysis of differentially expressed genes with selenium contents in the stem. **(A)** Hierarchical cluster tree showing 10 modules with coexpressed genes. **(B)** Module with selenium content correlations and the corresponding p-values. **(C–E)** Correlation between module membership and significance of gene related to selenium contents. **(F)** GO annotation of genes in green, brown, and blue modules. **(G)** Network analysis of hub genes in the stem.

After WGCNA analysis, 6,169 differentially expressed genes in the stem were classified into 10 modules (**Figure 4A**). The correlation analysis between 10 modules and selenium contents in stem showed a significant positive correlation between the MEblue module (967 genes) and selenium contents (r = 0.78, p = 0.0028) (**Figure 4B**). The MEbrown module (r = −0.97, p = 0.00000014) and the MEgreen module (r = −0.73, p = 0.0074) were negatively correlated with selenium contents having 621 and 308 genes, respectively. The selenium contents of four genotypes in the stem were determined in the order of 5097B > 2057B > YNSM > CH727. The expression level of genes in the MEblue module in 5097B was significantly higher than those of the other three genotypes (**Figure 2A**). In the MEbrown module, the expression levels of genes in high-selenium genotypes (2057B and 5097B) were significantly lower than those of the low-selenium genotypes (CH727 and YNSM) (**Supplementary Figure S2B**), and the expression levels of genes in MEgreen in 5097B were significantly lower than those of the other three genotypes (**Supplementary Figure S2C**). A significant correlation was observed between module membership (MM) and gene significance (GS) (**Figures 4C–E**). In addition, GO enrichment analysis of 1,896 genes in these three modules revealed that the GO classes that may be associated with selenium transport are as follows: GO:0035435: “phosphate ion transmembrane transport”; GO:1901682: “sulfur compound transmembrane transporter activity”; GO:0098656: “anion transmembrane transport”; GO:0006811: “ion transport”; GO:0000041: “transition metal ion transport” (**Figure 4F**; **Supplementary Table S4**).

**Figure 5:**
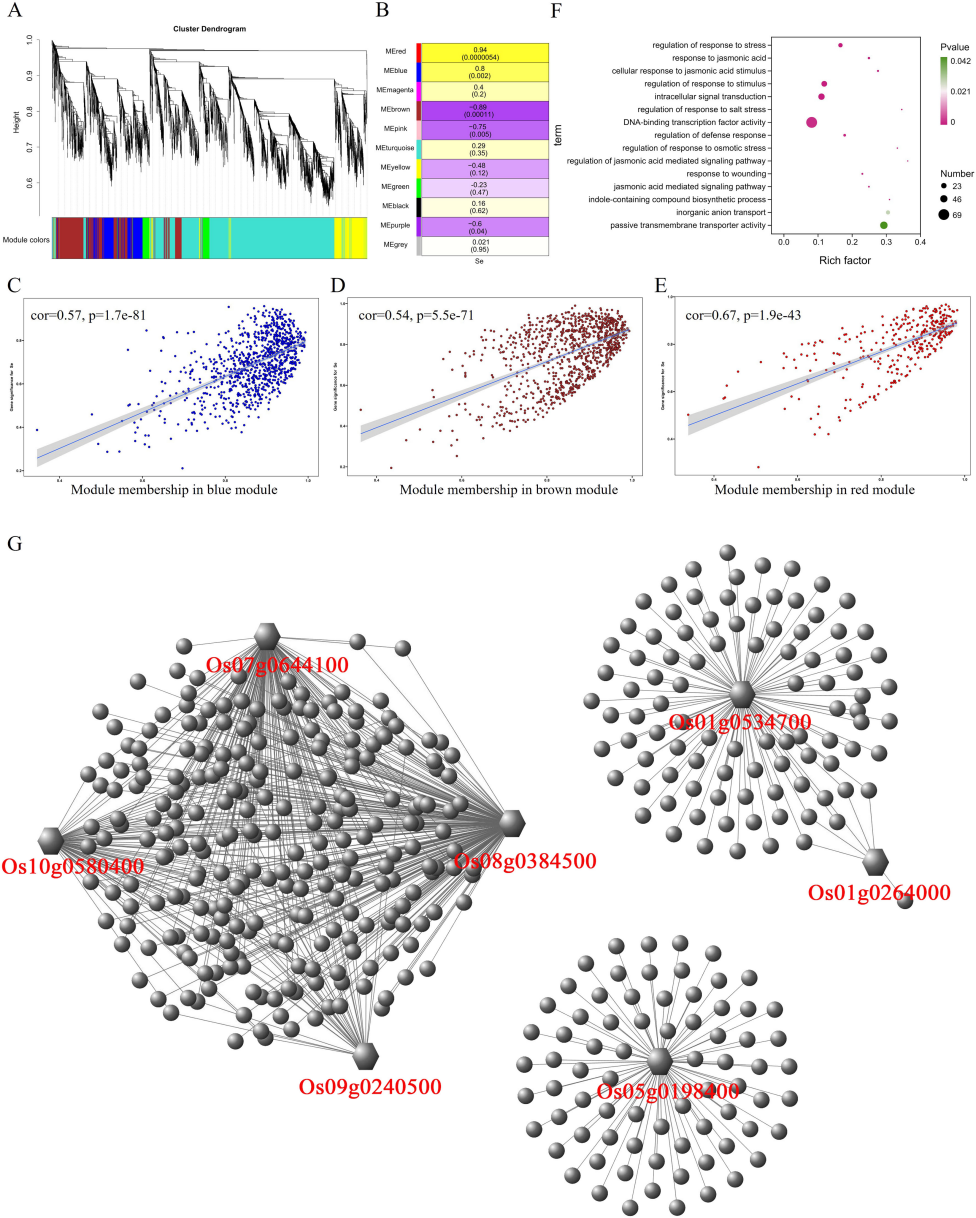
Weighted gene coexpression network analysis of differentially expressed genes with selenium contents in leaves. **(A)** Hierarchical cluster tree showing 11 modules with coexpressed genes. **(B)** Module with selenium content correlations and the corresponding p-values. **(C–E)** Correlation between module membership and significance of gene related to selenium contents. **(F)** GO annotation of genes in red, brown, and blue modules. **(G)** Network analysis of hub genes in leaf.

A total of 7,609 differentially expressed genes were identified in leaves, and 11 modules were obtained after the WGCNA of these 7,609 genes (**Figure 5A**). By analyzing the correlation between these 11 modules and selenium contents in leaves, it was found that MEred, MEblue, MEbrown, and MEpink were significantly correlated with selenium contents (p < 0.01) (**Figure 5B**). MEred (r = 0.94, p = 0.0000054) and MEblue (r = 0.8, p = 0.002) have significant positive correlations with selenium contents containing 323 and 933 genes, respectively. MEbrown (r = −0.89, p = 0.00011) and MEpink (r = −0.75, p = 0.005) were negatively correlated with selenium contents, containing 923 and 171 genes, respectively. The expression level of genes in MEblue and MEred modules were significantly lower in CH727 and YNSM than those in 2057B and 5097B (**Supplementary Figures S3A, C**). In the MEbrown module, the gene expression levels in the 2057B and 5097B genotypes were significantly lower than those of the CH727 and YNSM genotypes (**Supplementary Figure S3B**). A significant correlation was observed between the module membership (MM) and gene significance (GS) in the three modules (**Figures 5C–E**). GO enrichment analysis of genes in these modules showed that the GO classes that may be related to selenium contents are as follows: GO:0055085: “transmembrane transport”; GO:0022803: “passive transmembrane transporter activity” (**Figure 5F**; **Supplementary Table S5**).

**Figure 6:**
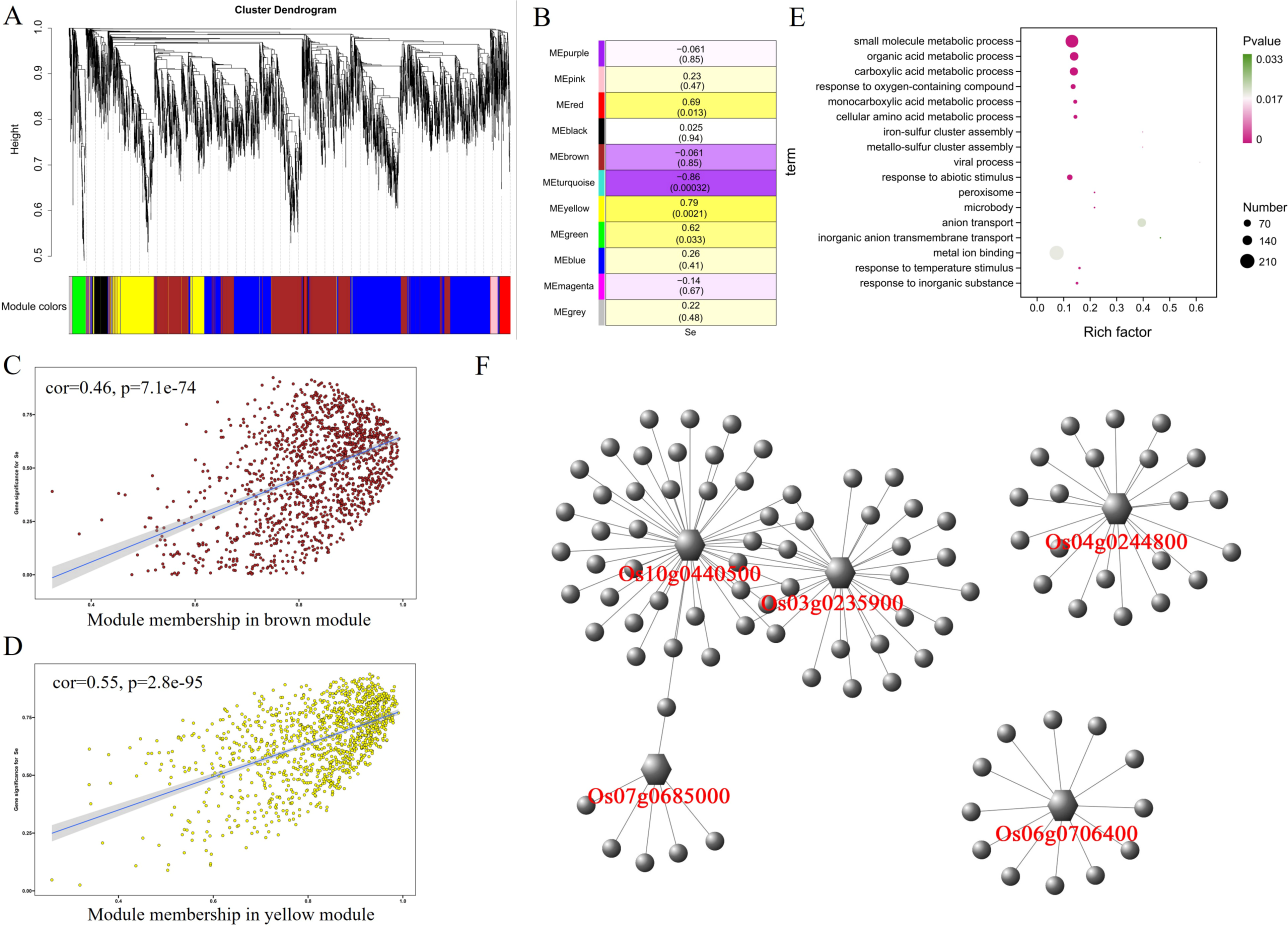
Weighted gene coexpression network analysis of differentially expressed genes with selenium contents in panicles. **(A)** Hierarchical cluster tree showing 11 modules with coexpressed genes. **(B)** Module with selenium content correlations and the corresponding p-values. **(C, D)** Correlation between module membership and significance of gene related to selenium contents. **(E)** GO annotation of genes in brown and yellow modules. **(F)** Network analysis of hub genes in panicles.

In panicles, 10,223 differentially expressed genes were divided into 11 modules (**Figure 6A**). After analyzing the correlation between these 11 modules and selenium contents, it was found that the MEyellow module having 1,193 genes had a significant positive correlation (r = 0.79, p = 0.0021) with selenium contents. The MEbrown module containing 1,393 genes was negatively correlated (r = −0.86, p = 0.00032) with selenium contents (**Figure 6B**). In the MEyellow module, the expression levels of genes in the 2057B and 5097B genotypes were significantly higher than those of the CH727 and YNSM genotypes (**Supplementary Figure S4A**). In the MEbrown module, the expression level of the gene in the 5097B was significantly lower than those of the other three genotypes (**Supplementary Figure S4B**). A significant correlation between module membership (MM) and gene significance (GS) was observed, i.e., MEbrown: r = 0.46, p = 7.1e−74; MEyellow: r = 0.55, p = 2.8e−95 (**Figures 6C, D**). GO enrichment analysis of genes in these modules revealed that the following GO classes might be related to selenium contents: GO:0098661: “inorganic anion transmembrane transport”; GO:0006820: “anion transport”; GO:0051540: “metal cluster binding” (**Figure 6E**; **Supplementary Table S6**).

### 3.4 Identification of genes involved in selenium uptake and translocation

**Figure 7:**
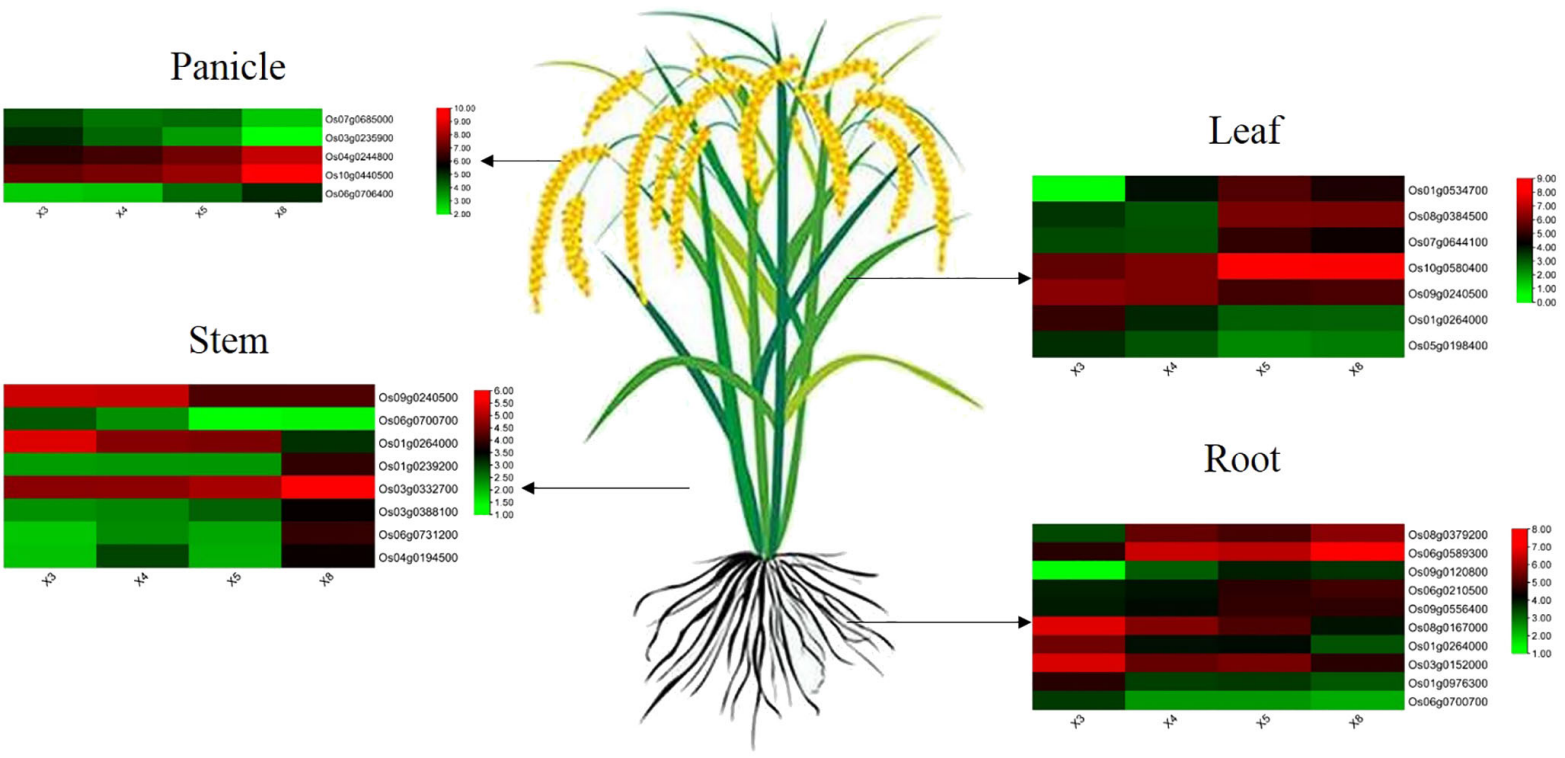
The expression pattern of hub genes involved in selenium uptake and translocation at the root, stem, leaf, and panicle in four genotypes.

**Figure 8:**
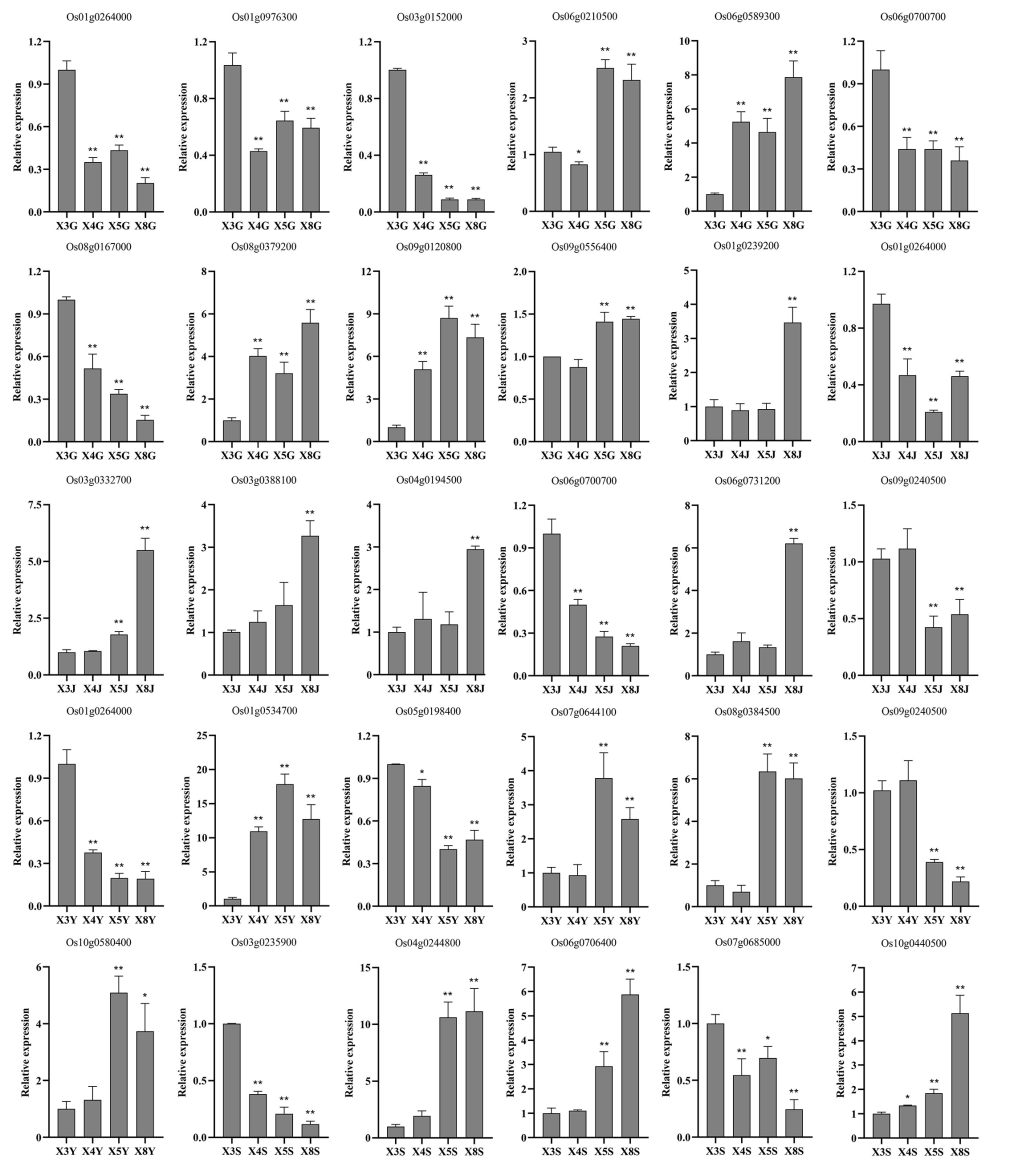
qRT-PCR analysis of the expression patterns of hub genes at the root, stem, leaf, and panicle in four genotypes. X3, X4, X5, and X8 represent CH727, YNSM, 2057B, and 5097B, respectively. G, J, Y, and S represent the root, stem, leaf and panicle, respectively. * and ** indicate significantly differential expression at 0.05 and 0.01 level.

After finding modules associated with selenium contents from differentially expressed genes detected by WGCNA from RNA-seq, we further screened core genes associated with selenium contents in each tissue from these modules. |MM| > 0.8, |GS| >0.8, and FPKM > 5 were used as thresholds to combine the functions of each gene to screen the core genes in the corresponding modules of each tissue. Twenty-seven core genes associated with selenium contents were screened in four tissues (**Figure 7**). These genes possess more edges and interact with many DEGs, which suggests their importance in the network (**Figures 3G**, **4G**, **5G**, **6F**). Among them, 10 are core genes in the roots as follows: four for ABC transporter protein (Os06g0589300, Os08g0167000, Os08g0379200, Os09g0120800), two for phosphate transmembrane transporter protein (Os06g0210500, Os09g0556400), one for DOF transcription factor (Os01g0264000), and three for heavy metal-transporting ATPase (Os01g0976300, Os03g0152000, Os06g0700700). Eight core genes were identified in stem: four for ABC transporters (Os03g0332700, Os03g0388100, Os06g0731200, Os04g0194500), one for sulfate transporter 4.1 (Os09g0240500), one for heavy metal-transporting ATPase (Os06g0700700), one for phosphate transporter (Os01g0239200), and one for DOF transcription factor (Os01g0264000). In leaves, seven core genes were screened: two for ABC transporters (Os01g0534700, Os08g0384500), two for ZIP transporters (Os05g0198400, Os07g0644100), one for DOF transcription factor (Os01g0264000), one for High-affinity urea transporter (Os10g0580400), and one for sulfate transporter 4.1 (Os09g0240500). Six core genes were screened in the panicles: three for heavy metal-transporting proteins (Os04g0244800, Os04g0390100, Os10g0440500), one for DOF transcription factor (Os07g0685000), and two for nitrate transporter proteins (Os03g0235900, Os06g0706400). Among them, DOF transcription factor (Os01g0264000) was identified as the core gene in rhizomes and leaves, heavy metal-transporting ATPase (Os06g0700700) was identified as the core gene in roots and stem, and sulfate transporter 4.1 (Os09g0240500) was identified as the core gene in stems and leaves. The results of the qPCR analysis of hub genes were consistent with the results of the transcriptome analysis (**Figure 8**).

## 4 Discussion

In this study, it was observed that the accumulation of selenium in the tissues of high- and low-selenium rice genotypes was very different. Selenium contents were highest in the roots, followed by the leaves, stems, and panicles. Previous studies also had similar trends confirming that selenium was highest in the roots, translocated differentially in different tissues, and lowest in the grains (Zhou et al., 2014; Zhang et al., 2019b). The roots are the main organs of plants that absorb nutrients and organic matter from the soil, so selenium contents in the roots are the highest among all other tissues, not only in rice but also in other plants such as wheat, maize, and buckwheat (Zhang et al., 2019b; Jiang et al., 2017; Han et al., 2013). Previous studies have shown that selenium contents in rice grains have significant genotypic differences among different genotypes, and high-selenium content genotypes can be used as selenium-rich carriers in selenium-deficient areas (Zhang et al., 2019b). From the grain-filling to the maturity stage, the selenium contents in the stem, leaves, and panicles of high-selenium rice were significantly higher than those of the low-selenium rice, but the selenium contents in roots were opposite suggesting that the differences in selenium contents in roots, stems, and leaves of different genotypes may be closely related to selenium transport and distribution capacity. The ability of rice to absorb and transport selenium varies with different genotypes and selenium transport capacity. High-selenium genotypes can promote selenium uptake and transport more efficiently from the root to the leaf than low-selenium rice genotypes (Liang et al., 2019). Previous studies have shown that the difference in grain selenium contents between different rice varieties depends on root absorption capacity and xylem transport capacity (Lu et al., 2013; Wu et al., 2015). Transcriptome sequence and WGCNA analysis were used to screen out some genes related to selenium contents and ABC transporter protein, heavy metal-transporting ATPase, phosphate transmembrane transporter protein, sulfate transporter 4.1, and nitrate transporter protein and DOF transcription factor were identified as potential genes.

In plants, ABC transporters use the hydrolysis of ATP to promote the transmembrane transport of solutes. It has been reported that phytochelatins and phytochelatin–heavy metal complexes in plants are involved in detoxifying heavy metals through the transmembrane transport of ABC-type transporters (Kretzschmar et al., 2011). Heavy metal-transporting ATPase (HMA) proteins are mainly involved in transporting metal ions. In *Arabidopsis*, overexpression of AtHMA4 showed high tolerance to heavy metals and increased transport of these metal ions from root to stem (Lekeux et al., 2019). It has been reported that the uptake of selenium by plants is mainly through phosphate transporters, and overexpression of phosphate transporter OsPT2 in rice significantly decreases selenite uptake by rice roots (Zhang et al., 2014). Like phosphate transporters, sulfate transporters (OsSultr) are also involved in selenium absorption and transport. Overexpression of OsSULTR1 significantly increased the selenate intake and the accumulation of sulfur and selenium in shoots and grains (Kumar et al., 2019). Nitrate transporter is not only involved in the transmembrane transport of nitrate but also involved in the transport of selenate, and overexpression of OsNRT1.1B can promote the transport of SeMet from roots to shoots and significantly increase the selenium contents in rice grains (Zhang et al., 2019a). In addition to these transporters, the transcription factor DOF was also identified as the core gene in rhizomes and leaves indicating its importance for the uptake and transport of selenium contents in rice plants. The DOF transcription factor increases the response of roots to ammonium salts by inducing the efficient absorption and accumulation of multiple nutrient ions in rice, which contributes to the increase in rice yield (Iwamoto and Tagiri, 2016). Functional analysis of these genes in *Arabidopsis thaliana* or rice showed that these genes play an important role in plant selenium uptake and transport. The role of these genes in selenium absorption, transport, and chelation in rice needs to be further analyzed.

Sulfate transporters mainly do the uptake of selenate by plant roots, and phosphate transporters mainly do the uptake of selenite (Wang et al., 2014). Selenate easily transferred from the roots to the shoots, without changing its composition; however, in leaves, it was reduced to selenite, converted to organic selenium compounds, and distributed to other plant tissues. Compared with selenate, selenite was easily converted into other forms after being absorbed by plant roots, such as selenium methionine and its oxides, selenomethylcysteine, etc., which were directly deposited in the roots, and only a very small share is transported to the aerial parts (Li et al., 2008). Asher et al. (1977) showed that when plants absorb selenate, that converted into selenite and other selenides and transported to the shoots and leaves, which converted into organic selenium. In this study, sulfate transporter genes were differentially expressed in the stem, leaves, and panicles but not in the roots (**Figure 7**). From transcriptome sequence analysis of high- and low-selenium genotypes, only phosphate transporter genes were identified as differentially expressed genes, not sulfate transporters. These results suggested that the difference in selenium contents between high- and low-selenium genotypes might be due to differences in selenite uptake by the roots from the soil through phosphate transporters.

## 5 Conclusions

In this study, we conducted transcriptome sequencing on two types of rice materials (low-selenium and selenium-rich rice) across roots, stems, leaves, and panicles to analyze the gene expression differences. Differentially expressed genes were observed in the roots, stems, leaves, and panicles. WGCNA was employed to pinpoint the key genes associated with selenium content and subsequently identified the core genes linked with selenium contents in the roots, stems, leaves, and panicles, respectively. The identification of these core genes substantially aids in advancing our understanding of the molecular mechanisms involved in selenium absorption and transport during the growth of rice and provides a genetic basis for selenium-rich rice breeding and might be instrumental in the future for genetic manipulation.

## Data Availability

The original contributions presented in the study are included in the article/**Supplementary Material**. Further inquiries can be directed to the corresponding authors.

## Supplementary material

Supplementary Figure 1 Expression pattern of genes in green, turquoise and blue modules in roots of four different genotypes.

Supplementary Figure 2 Expression pattern of genes in blue, brown and green modules in stems of four different genotypes.

Supplementary Figure 3 Expression pattern of genes in blue, brown and red modules in leaves of four different genotypes.

Supplementary Figure 4 Expression pattern of genes in brown and yellow modules in panicles of four different genotypes.

Supplementary Table 1 List of primers used in this study.

Supplementary Table 2 Summary of RNA-seq data for 48 samples.

Supplementary Table 3 Go terms significantly enriched in roots.

Supplementary Table 4 Go terms significantly enriched in stems.

Supplementary Table 5 Go terms significantly enriched in leaves.

Supplementary Table 6 Go terms significantly enriched in panicles.
