# Supplementary material for: Transcriptome analysis of high- and low-selenium genotypes identifies genes responsible for selenium absorption, translocation, and accumulation
Source: Front Plant Sci. 2024 Sep 23;15:1413549. doi: 10.3389/fpls.2024.1413549 (PMC11456430; doi:10.3389/fpls.2024.1413549)
Supplement: Supplementary Figure 1 — Expression pattern of genes in green, turquoise and blue modules in roots of four different genotypes. [file DataSheet1.doc]

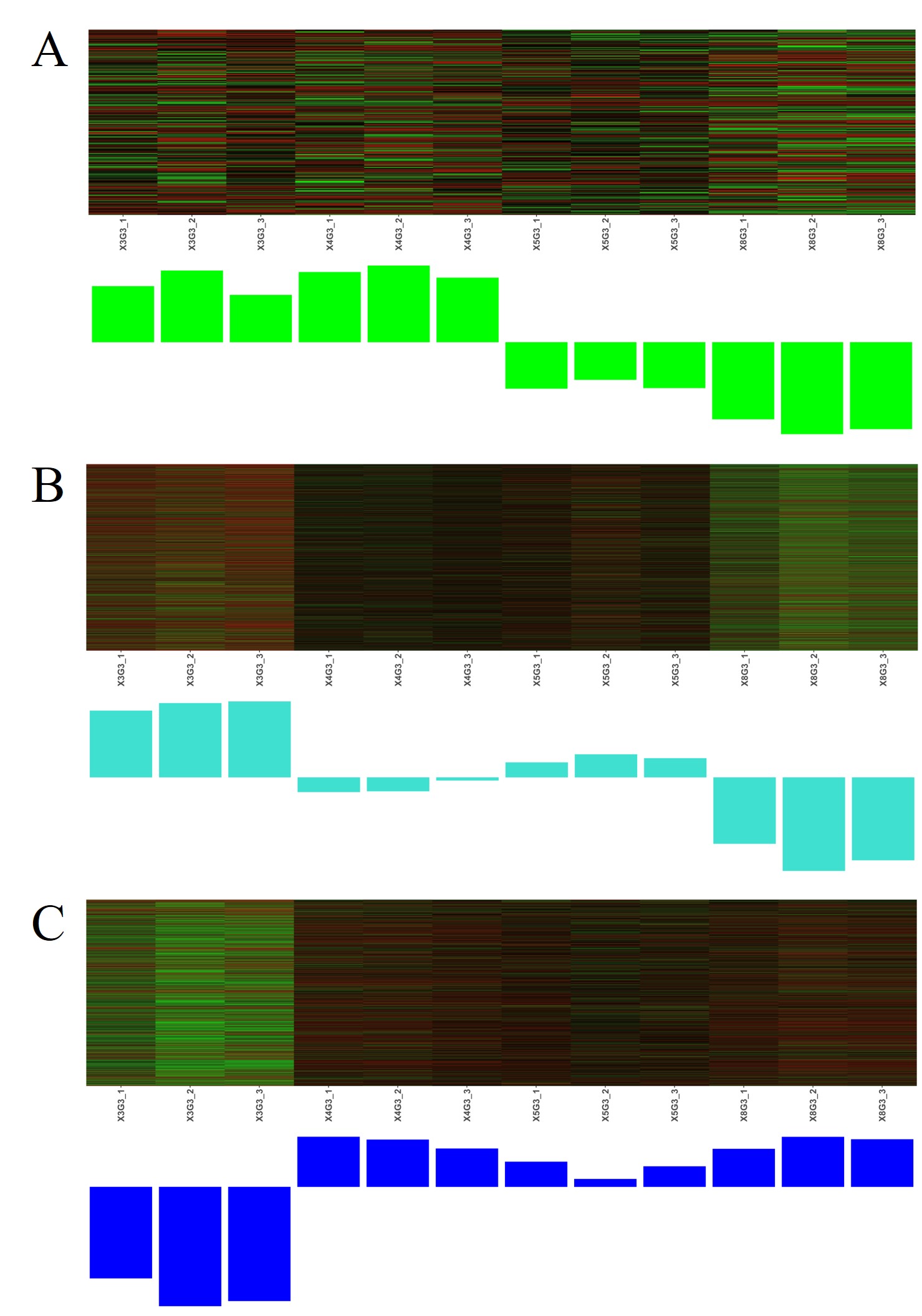


**Figure S1.** Expression pattern of genes in green, turquoise and blue modules in roots of four different genotypes.


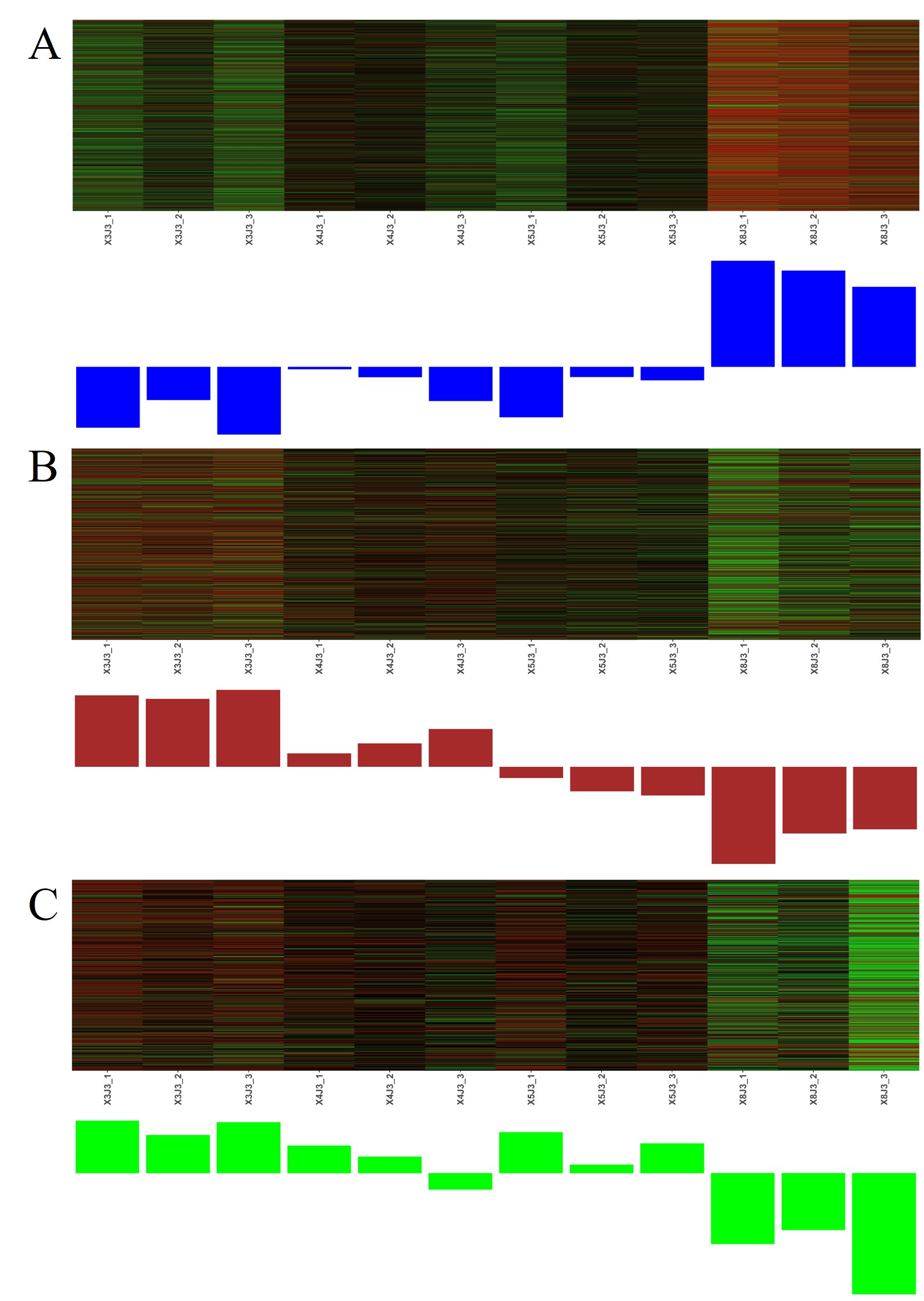


**Figure S2.** Expression pattern of genes in blue, brown and green modules in stems of four different genotypes.


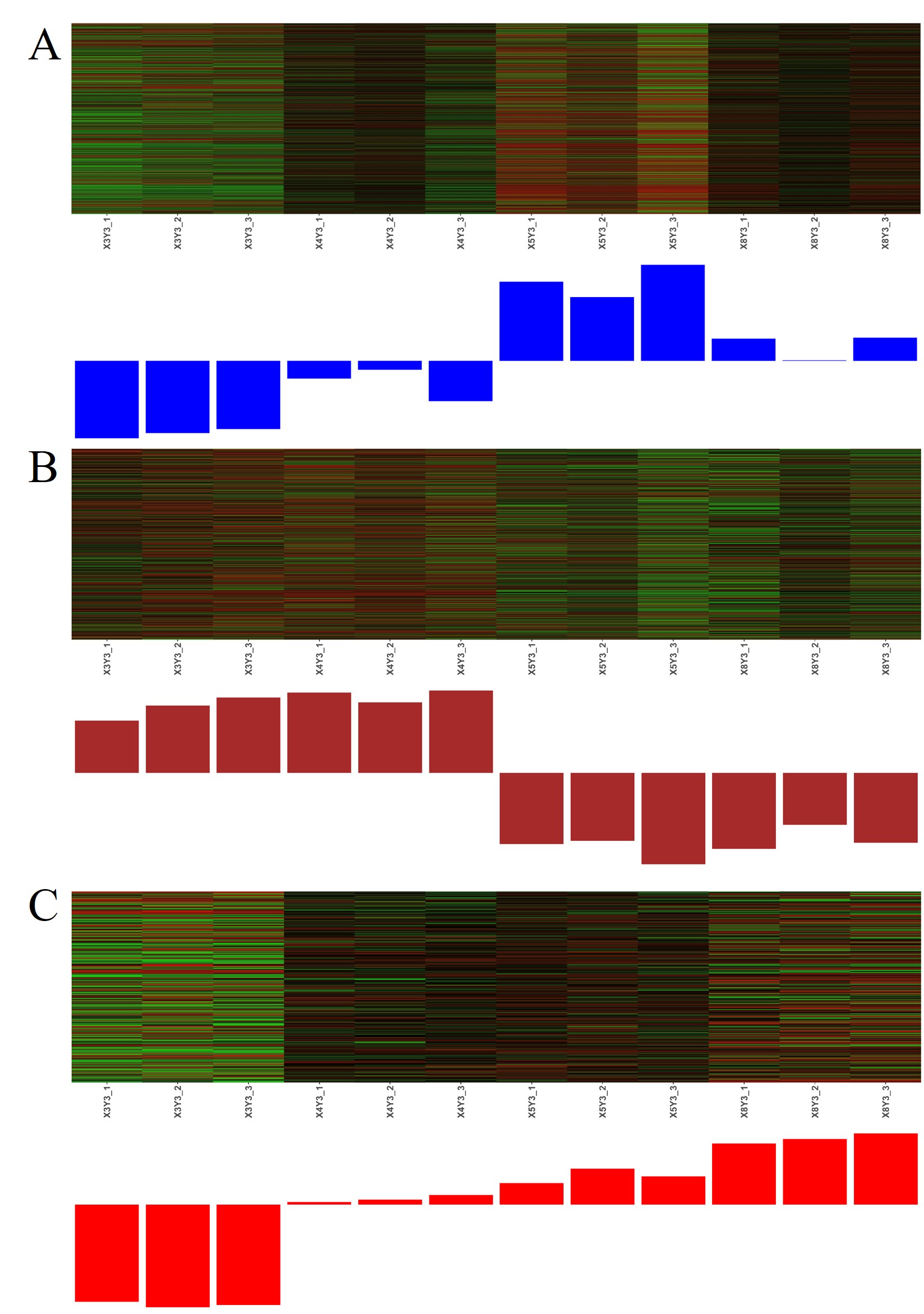


**Figure S3.** Expression pattern of genes in blue, brown and red modules in leaves of four different genotypes.


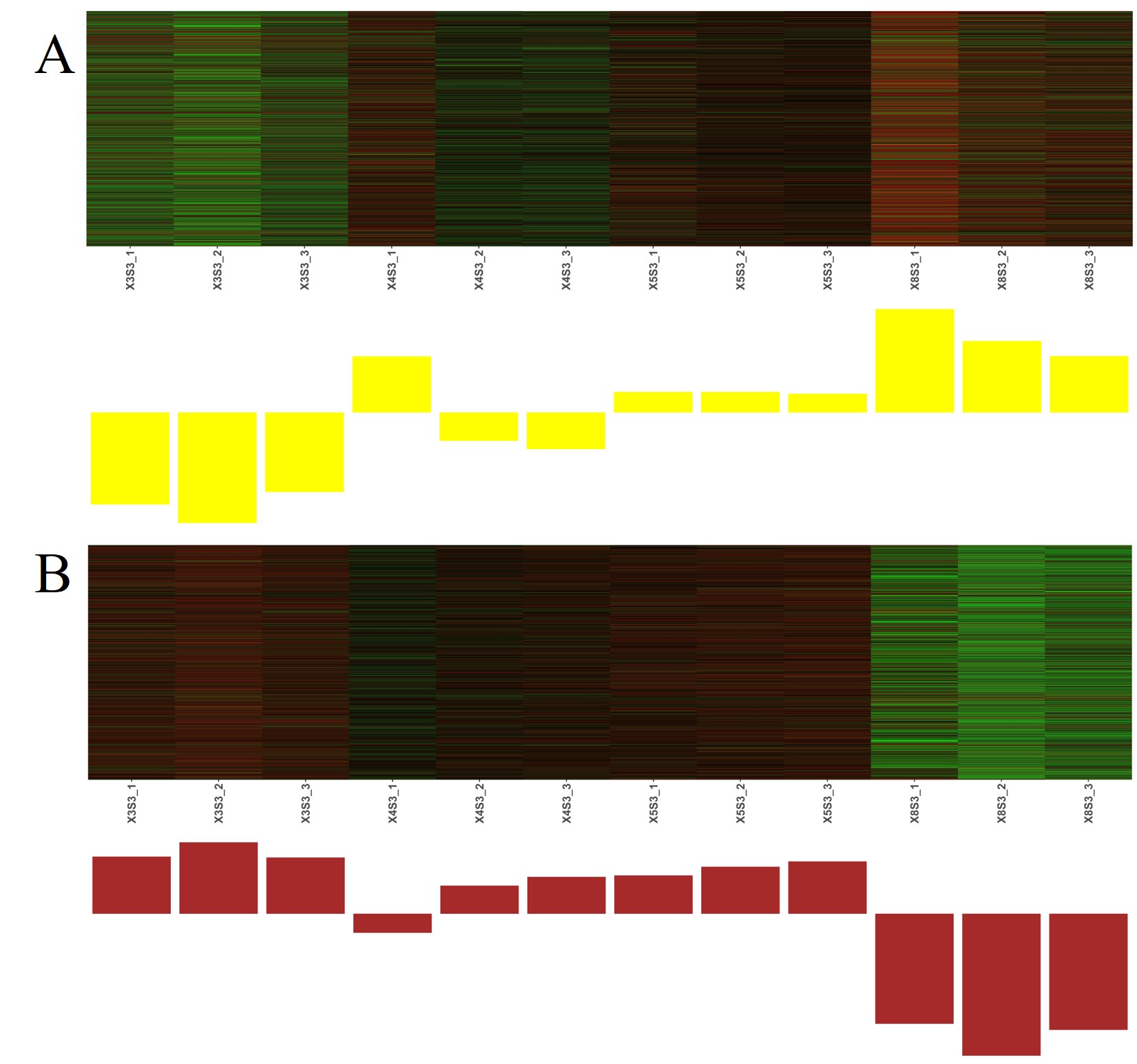


**Figure S4.** Expression pattern of genes in brown and yellow modules in panicles of four different genotypes.
